# Genotypes of carboxypeptidase A1 and gamma‐glutamyltransferase 1 may be useful tools for the diagnosis and the predictor of worrisome features of intraductal papillary mucinous neoplasm in Japan

**DOI:** 10.1002/jgh3.70031

**Published:** 2024-10-07

**Authors:** Shuhei Agawa, Seiji Futagami, Ken Nakamura, Mayu Habiro, Rie Kawawa, Yuto Shinagawa, Rina Motomiya, Kumiko Kirita, Teppei Akimoto, Takeshi Onda, Tomohide Tanabe, Nobue Ueki, Kazufumi Honda, Kok‐Ann Gwee, Katsuhiko Iwakiri

**Affiliations:** ^1^ Division of Gastroenterology, Department of Internal Medicine Nippon Medical School Tokyo Japan; ^2^ Department of Bioregulation Graduate School of Medicine, Nippon Medical School Tokyo Japan; ^3^ Department of Medicine, Yong Loo Lin School of Medicine National University of Singapore Singapore Singapore

**Keywords:** carboxypeptidase A1, endosonography, gamma‐glutamyltransferase 1, intraductal papillary mucinous neoplasm, single‐nucleotide polymorphism

## Abstract

**Background and Aim:**

This study aimed to clarify whether several single‐nucleotide polymorphisms (SNPs)‐related chronic pancreatitis such as carboxypeptidase A1 (*CPA1*), carboxypeptidase B1 (*CPB1*), Gamma‐glutamyltransferase 1 (*GGT1*), G‐protein‐coupled receptor Class C Group 6 Member A (*GPRC6A*), and serine protease inhibitor, Kazal type 1 (*SPINK‐1*) genotypes were associated with clinical characteristics of patients with intraductal papillary mucinous neoplasm (IPMN) and worrisome features of IPMN.

**Methods:**

We enrolled 100 patients with IPMN and 116 patients as a control. Serum p‐amylase, lipase, trypsin, phospholipase A2 (PLA2), and elastase‐1 levels were measured. An Olympus EUS (GF‐UCT 260) was used to perform endosonography in 100 patients with IPMN. Total EUS score was evaluated using endosonography. DNA was isolated from the duodenal tissue using a commercial system and polymerase chain reaction (PCR) was performed on 7500 Fast PCR System.

**Results:**

There were no associations between glucose tolerances, lipid levels and genotypes of *CPA1*, *GGT1*, *GPRC6A*, and *SPINK‐1* in patients with IPMN. *CPA1* genotype was significantly associated with the pathophysiology of IPMN. Then, *GGT1* genotype was also significantly associated with EUS total score and the size of cyst more than 20 mm and more than 30 mm as one of worrisome features of IPMN.

**Conclusion:**

Genotypes of carboxypeptidase A1 and gamma‐glutamyltransferase 1 may be useful tools for the diagnosis and the predictor of worrisome features of IPMN.

## Graphical abstract

*CPA1* and *GGT1*, a type of SNP, may be associated with intraductal papillary mucinous neoplasm (IPMN). In this study, *CPA1* and *GGT1* were associated with the presence of IPMN and cyst size, respectively. It may be useful tools for the diagnosis and the predictor of worrisome features of intraductal papillary mucinous neoplasm in Japan.

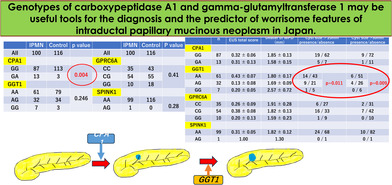

## Introduction

Pancreatic cancer is known as a disease with poor prognosis; early detection and early treatment are effective way to reduce the mortality from the disease. High‐risk diseases of pancreatic cancer include chronic pancreatitis and intraductal papillary mucinous neoplasm (IPMN). It is known that 2–10% of branch duct‐IPMN have the potential to become malignant, and high‐risk stigmata and worrisome features are findings that suggest malignant transformation. Although the rate of malignant transformation is low, guidelines recommend continuous follow‐up is owing to the risk of malignant transformation. Therefore, several patients with IPMN undergo abdominal CT or MRI annually. Therefore, the risk of potential malignancy in patients with IPMN should be evaluated using noninvasive examinations. Several studies have reported that lifestyle and genetic factors play a role in the transformation of pancreatic ductal cancer.^1^, ^2^, ^3^, ^4^, ^5^ The mutations of *KRAS* and *GNAS* are frequently observed in IPMN patients.^6^ Furthermore, several genetic associations with chronic pancreatitis have been identified in the complex pathophysiology of pancreatic diseases.^7^ Most of the associated genes induced pancreatitis within pancreatic acinar cells via a trypsin‐dependent pathway such as the cationic trypsinogen gene (Protease serine 1: *PRSS1*), serine protease inhibitor, Kazal type 1 (*SPINK‐1*), and Chymotrypsin C (*CTRC*)^7^, ^8^, ^9^ or misfolding‐dependent pathway including carboxypeptidase A1 (*CPA1*)^10^, ^11^ and carboxypeptidase B1 (*CPB1*).^12^ Kaune et al. reported the relationship between G‐protein‐coupled receptor Class C Group 6 Member A (*GPRC6A*) variants and pancreatitis etiologies,^13^ and Diergaarde et al. have reported the association between Gamma‐glutamyltransferase 1 (*GGT1*) gene in pancreatic carcinogenesis.^14^ Although endosonography frequently reveals the evidence of chronic pancreatitis (CP) in the background pancreatic parenchyma of patients with IPMN,^15^ there were no available data about the association between SNP‐related CP and clinical characteristics of patients with IPMN.

Furthermore, we have also reported that the detection of the cleaved pattern of the C‐terminal ends of the amino acids of the apolipoprotein A2 homodimer can distinguish patients with stage I/II pancreatic cancer and associated diseases from healthy subjects.^16^, ^17^, ^18^ Hayasaki et al. have demonstrated that the clinical significance of apoA2 isoforms, AT, and ATQ levels in patients with pancreatic cancer compared with alterations in pancreatic morphology, such as the diameter of the main pancreatic duct or pancreatic parenchymal volume as markers of pancreatic exocrine dysfunction.^19^ Additionally, we have first reported that apolipoprotein A2 isoform was significantly associated with exocrine pancreatic insufficiency by multiple logistic regression analysis.^20^ Therefore, we aimed to investigate the role of *CPA1* in chronic pancreatitis. Digestive carboxypeptidases are pancreatic metalloproteases that hydrolyze the C‐terminal peptide bonds in dietary polypeptide chains. *CPA1* variants with less than 20% apparent activity were overrepresented in the chronic pancreatitis group compared with that in controls.^21^

This study aimed to clarify whether several SNP‐related chronic pancreatitis such as *CPA1*, *CPB1*, *GGT1*, *GPRC6A*, and *SPINK‐1* genotypes were associated with clinical characteristics of patients with IPMN and stigmata of IPMN.

## Methods

### Patients

We enrolled 100 patients with IPMN and 116 patients as a control. The IPMN (Branch duct‐IPMN:BD‐IPMN) group included patients who visited Nippon Medical School Musashi Kosugi Hospital and Nippon Medical School Hospital between February 2019 and January 2023, were diagnosed using MRI, and underwent endosonography. The study protocol was approved by the Ethics Review Committee (M‐2023‐112) of the Nippon Medical School Hospital. Written informed consent was obtained from all enrolled patients after the approval by the Ethical Review Committee. In addition, the study protocol conforms to the ethical guidelines of the 1975 Declaration of Helsinki as reflected in a priori approval by the institution's human research committee. Our study did not include minors. The exclusion criteria were severe heart disease, renal or pulmonary failure, liver cirrhosis, severe diabetes mellitus, severe systemic illness, a history of malignant disease.

### Definition of pancreatic enzyme abnormalities

Serum p‐amylase, lipase, trypsin, phospholipase A2 (PLA2) and elastase‐1 levels were measured using the same automated chemistry analyzer (AU 5822 analyzer; Beckman Coulter, Brea, CA, USA). The validated reference ranges of pancreatic enzymes at our hospital were 18–53 (U/L) for p‐amylase, 11–53 (U/L) for lipase, 100–550 (ng/mL) for trypsin, 130–400 (ng/dL) for PLA2, and 0–300 (ng/dL) for elastase‐1.

### Endosonographic assessment

An Olympus EUS (GF‐UCT 260, Olympus, Tokyo, Japan) was used to perform endosonography (EUS) in 100 patients with IPMN under conscious sedation with the agreement. Data of EUS total score represent the sum of the score of lobularity, standing or hyperechoic foci, hyperechoic main pancreatic duct (MPD) and dilated side branches.^22^ Two or more endoscopists evaluated the presence or absence of the four items listed in the 2019 Chronic Pancreatitis Diagnostic as endpoints of microinflammation in the pancreas. If differences were found in opinion among specialist endoscopists, the final diagnosis was determined by consensus after each case was discussed during the procedure of EUS.

### The dilater of MPD and size of cyst in IPMN

To evaluate IPMN, the Revisions of International Consensus Fukuoka guidelines^23^ for the management of IPMN of the pancreas were used as reference, and the MPD diameter, cyst diameter of the IPMN, based on the endosonographic features, and presence of internal nodules were assessed. MPD was measured in pancreatic tail using endosonography.

### DNA extraction and SNP genotyping

DNA was isolated from the duodenal tissue from duodenal second portion using a commercial system (AllPrep DNA/RNA Mini Kit, Qiagen, Hilden, Germany). Polymerase chain reaction (PCR) was performed on 7500 Fast PCR System (Thermo Fisher Scientific, MA, USA) under the following cycle conditions, pre‐read stage; 60°C for 1 min, hold stage; 95°C for 10 min, followed 40 cycles of 95°C for 15 s and 60°C for 1 min, post read stage; 60°C for 1 min. TaqMan genotyping reactions were carried out in a total volume of 10 μL containing 1x TaqMan™ Genotyping Master Mix (Thermo Fisher Scientific, MA, USA), 1x primer (rs4820599 of *GGT1*, rs1606365 of *GPRC6A*, rs17107315 of *SPINK1*, rs184981267 and rs201750163 of *CPA1*, rs141911824 and rs201519774 of *CPB1* were ready‐made primers, rs77792157 of *CPA1* was custom‐made primer, Thermo Fisher Scientific, MA, USA) and 10‐ng DNA. For each analysis, artificially synthesized positive controls were prepared as well.

### Sample size

The sample size was determined using G* Power 3.1.9.7 (Faul, Erdfelder, Lang, & Buchner). Distributions of *CPA1*(rs77792157), genotypes were 113GG (97.4%), 3GA (2.6%) in control, 87GG (87%), 13GA (13%) in IPMN group. Using the above data, and setting α = 0.05, 1‐β = 0.80, it was determined that the effect size was 0.309, 83 patients were estimated to be sufficient to identify clinically relevant differences.

### Statistical analysis

Data were evaluated using two‐tailed unpaired t‐tests for continuous variables of *CPA1* and one‐way analysis of variance (ANOVA), followed by post hoc testing with Bonferroni correction for continuous variables of *GPRC6A* and *GGT1*. Categorical variables were compared using the chi‐square (χ2) test. All analyses were performed using SPSS (version 27.0; IBM Corp., Armonk, NY, USA), and statistical significance was set at *P* < 0.05.

## Results

### Clinical characteristics of patients with IPMN

Table 1 shows clinical characteristics of patients with IPMN. The mean age of the IPMN and control groups was 65.3 ± 1.2 and 62.7 ± 1.4 years, respectively, and gender (M/F) was 44/56 and 45/71, with no significant difference between the two group (Table 1).

**Table 1:** Clinical characteristics of patients with IPMN

*n*	100	T‐Cho (mg/dL)	209.8 ± 4.9
Age	65.3 ± 1.2	LDL‐Cho (mg/dL)	123.6 ± 3.4
Gender M/F	44/56	HDL‐Cho (mg/dL)	65.1 ± 2.0
BMI	22.6 ± 0.4	TG (mg/dL)	117.9 ± 7.2
Smoking (BI)	115.9 ± 23.6	P‐amylase (%)	22.7
Alcohol (g/day)	9.3 ± 2.4	Lipase (%)	23.5
BS (mg/dL)	108.1 ± 2.7	Trypsin (%)	31.6
HbA1c (%)	6.0 ± 0.1	PLA2 (%)	20.4
Insulin (μU/mL)	11.8 ± 1.1	Elastase‐1 (%)	8.6

Data of pancreatic enzyme represent the percentage of patients with abnormal values. Data are represented as mean value ± SEM.

BI, Brinkman Index; BMI, body mass index; BS, blood sugar; HbA1c, hemoglobin A1c; HDL‐Cho, high density lipoprotein cholesterol; LDL‐Cho, low‐density lipoprotein cholesterol; PLA2, phospholipase A2; T‐Cho, total cholesterol; TG, triglyceride.

### Distribution of genotypes between the control and IPMN group

Distributions of *CPA1*(rs77792157), *GGT1*, *GPRC6A*, and *SPINK‐1* genotypes in control group were 113GG(97.4%), 3GA(2.6%); 79AA(68.1%), 34AG(29.3%), 3GG(2.6%); 43CC(37.1%), 55CG(47.4%), 18GG(15.5%); 116AA(100%), respectively (Table 2). The distributions of *CPA1*(rs77792157), *GGT1*, *GPRC6A*, and *SPINK‐1* genotypes in the IPMN group were 87GG (87%), 13GA (13%); 61AA (61%), 32AG (32%), 7GG (7%); 35CC (35%), 54CG (54%), 10GG (10%); 99AA (99%), 1AG (1%), respectively. There was a significant difference (*P* = 0.004) in distribution of *CPA1*(rs77792157) between the IPMN and control group. No genotypic variation was observed in rs184981267 and rs201750163 of *CPA1*, rs141911824 and rs201519774 of *CPB1*; thus, *CPA1* represents rs77792157 below.

**Table 2:** Distribution of each genotype between IPMN and control group

	IPMN	Control	*P* value
All	100	116	
CPA1			
*GG*	87	113	0.004
*GA*	13	3	
GGT1			
*AA*	61	79	0.246
*AG*	32	34	
*GG*	7	3	
GPRC6A			
*CC*	35	43	0.410
*CG*	54	55	
*GG*	10	18	
SPINK1			
*AA*	99	116	0.280
*AG*	1	0	

CPA1, carboxypeptidase A1; GGT1, gamma‐glutamyltransferase 1; GPRC6A, G‐protein‐coupled receptor class C Group 6 member A; IPMN, intraductal papillary mucinous neoplasm; SPINK1, serine protease inhibitor, Kazal type 1.

### Clinical characteristics of each genotype in patients with IPMN

Each genotype of *CPA1*, *GGT1*, *GPRC6A*, and *SPINK‐1* in patients with IPMN was not significantly associated with age, sex, body mass index (BMI), smoking, or alcohol consumption (Table 3).

**Table 3:** Clinical characteristics of each genotype in patients with IPMN

	*n*	Age	Gender M/F	BMI	Smoking (BI)	Alcohol (g/day)	*P*1 value	*P*2 value	*P*3 value	*P*4 value	*P*5 value
All	100										
CPA1											
*GG*	87	65.0 ± 1.4	38/49	22.5 ± 0.4	97.2 ± 22.8	7.8 ± 2.0	0.396	0.867	0.476	0.163	0.369
*GA*	13	68.1 ± 2.9	6/7	23.3 ± 1.3	243.3 ± 95.9	18.7 ± 11.6					
GGT1											
*AA*	61	65.1 ± 1.6	28/33	22.9 ± 0.5	119.0 ± 30.5	11.4 ± 3.5	1.000	0.682	0.822	1.000	1.000
*AG*	32	65.2 ± 2.1	14/18	22.0 ± 0.7	131.4 ± 44.7	6.6 ± 2.5	1.000		1.000	0.726	1.000
*GG*	7	68.9 ± 4.6	2/5	20.8 ± 0.4	10.0 ± 10.0	0.4 ± 0.4	1.000		0.984	0.814	0.925
GPRC6A											
*CC*	35	65.2 ± 2.1	13/22	22.4 ± 0.7	73.7 ± 25.3	4.4 ± 1.9	1.000	0.577	1.000	0.498	0.214
*CG*	54	65.7 ± 1.6	26/28	22.7 ± 0.5	143.1 ± 38.7	13.7 ± 4.1	1.000		1.000	1.000	0.625
*GG*	10	65.2 ± 5.1	4/6	22.1 ± 0.8	75.0 ± 49.7	2.0 ± 2.0	1.000		1.000	1.000	1.000
SPINK1											
*AA*	99	65.3 ± 1.2	43/56	22.6 ± 0.4	115.4 ± 23.6	9.3 ± 2.4		0.257			
*AG*	1	77	1/0	24.5	160.0	14.0					

*P*1 value: *P* value of age, *P*2 value: *P* value of gender, *P*3 value: *P* value of BMI, *P*4 value: *P* value of smoking, *P*5 value: *P* value of alcohol. For continuous variables of GGT1, *P* value of AA vs. AG is shown in the top row, AG vs. GG in the second row, GG vs. AA at the bottom. For continuous variables of GPRC6A, *P* value of CC vs. CG is shown in the top row, CG vs. GG in the second row, GG vs. CC at the bottom. Statistical analysis for continuous variables of SPINK1 were not possible because the number of AG was too small. Data are represented as mean value ± SEM.

BI, Brinkman Index; BMI, body mass index; CPA1, carboxypeptidase A1; GGT1, gamma‐glutamyltransferase 1; GPRC6A, G‐protein‐coupled receptor class C Group 6 member A; IPMN, intraductal papillary mucinous neoplasm; SPINK1, serine protease inhibitor Kazal type 1.

### Comparison of the associations between glucose tolerances and genotypes of CPA1, GGT1, GPRC6A, and SPINK‐1 in patients with IPMN

To investigate glucose tolerances for each genotype in patients with IPMN, we compared the blood sugar level, HbA1c, and serum insulin between the groups. However, each genotype of *CPA1*, *GGT1*, *GPRC6A*, and *SPINK‐1* in patients with IPMN was not significantly associated with them (Table 4).

**Table 4:** Comparison of glucose tolerances of each genotype in patients with IPMN

	*n*	BS (mg/dL)	HbA1c (%)	Insulin (μU/mL)	*P*1 value	*P*2 value	*P*3 value
All	100						
CPA1							
*GG*	87	108.0 ± 3.1	6.0 ± 0.1	11.2 ± 1.0	0.898	0.307	0.137
*GA*	13	108.8 ± 5.3	5.8 ± 0.2	16.2 ± 5.4			
GGT1							
*AA*	61	107.5 ± 3.0	6.0 ± 0.1	13.5 ± 1.6	1.000	1.000	0.254
*AG*	32	104.3 ± 5.3	6.0 ± 0.2	9.3 ± 1.4	0.069	1.000	1.000
*GG*	7	130.3 ± 15.	6.1 ± 0.2	7.9 ± 1.7	0.110	1.000	0.718
GPRC6A							
*CC*	35	108.5 ± 4.6	6.1 ± 0.2	11.4 ± 1.5	1.000	0.983	1.000
*CG*	54	109.0 ± 3.9	5.9 ± 0.1	12.8 ± 1.7	0.723	1.000	0.542
*GG*	10	97.9 ± 6.0	5.8 ± 0.1	7.4 ± 2.3	0.853	1.000	1.000
SPINK1							
*AA*	99	108.2 ± 2.8	6.0 ± 0.1	11.8 ± 1.1			
*AG*	1	97.0	5.7	6.4			

*P*1 value: *P* value of blood sugar, *P*2 value: *P* value of HbA1c, *P*3 value: *P* value of insulin. For GGT1, *P* value of AA vs. AG is shown in the top row, AG vs. GG in the second row, GG vs. AA at the bottom. For GPRC6A, *P* value of CC vs. CG is shown in the top row, CG vs. GG in the second row, GG vs. CC at the bottom. Statistical analysis for continuous variables of SPINK1 were not possible because the number of AG was too small. Data are represented as mean value ±SEM.

BS, blood sugar; CPA1, carboxypeptidase A1; GGT1, gamma‐glutamyltransferase 1; GPRC6A, G‐protein‐coupled receptor class C Group 6 member A; HbA1c, hemoglobin A1c; IPMN, intraductal papillary mucinous neoplasm; SPINK1, serine protease inhibitor Kazal type 1.

### Comparison of serum lipid levels of each genotype in patients with IPMN

The genotypes *CPA1*, *GGT1*, *GPRC6A*, and *SPINK‐1* in patients with IPMN were not significantly associated with serum lipid levels, such as T‐Cho, LDL‐Cho, HDL‐Cho, and triglyceride (Table 5).

**Table 5:** Comparison of blood lipids of each genotype in patients with IPMN

	*n*	T‐Cho (mg/dL)	LDL‐Cho (mg/dL)	HDL‐Cho (mg/dL)	TG (mg/dL)	*P*1 value	*P*2 value	*P*3 Value	*P*4 value
All	100								
CPA1									
*GG*	87	209.3 ± 5.5	124.0 ± 3.8	64.4 ± 2.0	119.4 ± 7.9	0.801	0.738	0.292	0.599
*GA*	13	213.0 ± 6.9	120.5 ± 8.0	71.2 ± 9.5	108.0 ± 17.1				
GGT1									
*AA*	61	210.3 ± 6.6	121.6 ± 4.6	65. ± 3.1	121.7 ± 10.1	1.000	1.000	1.000	1.000
*AG*	32	208.3 ± 8.0	125.3 ± 5.8	65.5 ± 2.4	114.6 ± 11.4	1.000	1.000	1.000	1.000
*GG*	7	212.5 ± 15.8	132.3 ± 10.5	62.8 ± 5.1	99.8 ± 10.5	1.000	1.000	1.000	1.000
GPRC6A									
*CC*	35	212.5 ± 10.1	127.4 ± 6.2	63.8 ± 3.6	131.5 ± 14.8	1.000	1.000	1.000	0.479
*CG*	54	208.8 ± 5.9	121.8 ± 4.7	66.4 ± 2.7	110.3 ± 7.5	1.000	1.000	1.000	1.000
*GG*	10	201.9 ± 4.9	118.0 ± 9.4	64.4 ± 6.5	91.7 ± 19.0	1.000	1.000	1.000	0.356
SPINK1									
*AA*	99	210.2 ± 4.9	123.78 ± 3.5	65.2 ± 2.0	118.2 ± 7.2				
*AG*	1	169	106	63	92				

*P*1 value: *P* value of T‐Cho, *P*2 value: *P* value of LDL‐Cho, *P*3 value: *P* value of HDL‐Cho, *P*4 value: *P* value of TG. For GGT1, *P* value of AA vs. AG is shown in the top row, AG vs. GG in the second row, GG vs. AA at the bottom. For GPRC6A, *P* value of CC vs. CG is shown in the top row, CG vs. GG in the second row, GG vs. CC at the bottom. Statistical analysis for SPINK1 were not possible because the number of AG was too small. Data are represented as mean value ± SEM.

CPA1, carboxypeptidase A1; GGT1, gamma‐glutamyltransferase 1; GPRC6A, G‐protein‐coupled receptor class C Group 6 member A; HDL‐Cho, high‐density lipoprotein cholesterol; IPMN, intraductal papillary mucinous neoplasm; LDL‐Cho, low‐density lipoprotein cholesterol; SPINK1, serine protease inhibitor Kazal type 1; T‐Cho, total cholesterol; TG, triglyceride.

### Comparison of the associations between the ratio of pancreatic enzyme abnormalities and genotypes of CPA1, GGT1, GPRC6A, and SPINK‐1 in patients with IPMN

The genotype *CPA1*, *GGT1*, *GPRC6A*, and *SPINK‐1* in patients with IPMN were not significantly associated with pancreatic enzyme abnormalities (Table 6).

**Table 6:** Prevalence of pancreatic enzyme abnormalities in patients with each genotype in patients with IPMN

	*n*	P‐amylase (%)	Lipase (%)	Trypsin (%)	PLA2 (%)	Elastase‐1 (%)	*P*1 value	*P*2 value	*P*3 Value	*P*4 value	*P*5 value
All	100										
CPA1											
*GG*	87	20.8	23.3	29.1	19.8	6.7	0.249	0.894	0.144	0.674	0.163
*GA*	13	36.4	25.0	50.0	25.0	20.0					
GGT1											
*AA*	61	19.2	25.0	33.3	20.0	8.7	0.115	0.670	0.434	0.158	0.701
*AG*	32	22.6	18.8	25.0	15.6	5.6					
*GG*	7	60.0	33.3	50.0	50.0	16.7					
GPRC6A											
*CC*	35	23.5	31.4	42.9	22.9	11.5	0.849	0.136	0.133	0.739	0.671
*CG*	54	23.9	22.6	28.3	20.8	7.7					
*GG*	10	14.3	0.0	11.1	11.1	0.0					
SPINK1											
*AA*	99	23.0	23.7	32.0	20.6	8.6	0.585	0.578	0.494	0.611	
*AG*	1	0.0	0.0	0.0	0.0	Not measurable					

Data represent the percentage of patients with abnormal values. *P*1 value: *P* value of p‐amylase, *P*2 value: *P* value of lipase, *P*3 value: *P* value of trypsin, *P*4 value: *P* value of PLA2, *P*5 value: *P* value of elastase‐1. Statistical analysis for elastase‐1 of SPINK1 was not possible because the data of AG were not measurable. Data are represented as mean value ±SEM.

CPA1, carboxypeptidase A1; GGT1, gamma‐glutamyltransferase 1; GPRC6A, G‐protein‐coupled receptor class C Group 6 member A; IPMN, intraductal papillary mucinous neoplasm; PLA2, phospholipase A2; SPINK1, serine protease inhibitor Kazal type 1.

### Comparison of EUS features with each genotype such as CPA1, GGT1, GPRC6A, and SPINK‐1 in patients with IPMN

EUS total score of the patients in *GGT1 AA* was significantly higher than *GGT1 AG* (Table 7). A cyst diameter of 30 mm or larger is considered a high‐risk factor for malignant transformation of IPMN. In the present study, in addition to the presence or absence of cysts larger than 30 mm, we also examined the presence or absence of cysts larger than 20 mm based on the endosonographic features. The number of patients in IPMN with the size of the cyst more than 20 and 30 mm were significantly associated with the genotype of *GGT1* (*P* = 0.011 and *P* = 0.09, respectively). There were no significant relationships between *CPA1*, *GPRC6A*, and *SPINK1* genotypes and the diameter of MPD, the size of cyst more than 20 mm and 30 mm in patients with IPMN.

**Table 7:** Comparison of EUS findings for each genotype with the dilater of MPD and the size of cyst in IPMN

	*n*	EUS total score	Dilater of MPD (mm)	Cyst size >20 mm presence/absence	Cyst size >30 mm presence/absence	*P*1 value	*P*2 value	*P*3 value	*P*4 value
All	100	0.32 ± 0.05	1.81 ± 0.12	24/69	10/83				
CPA1									
*GG*	87	0.32 ± 0.06	1.85 ± 0.13	19/62	9/72	0.094	0.473	0.373	0.890
*GA*	13	0.31 ± 0.13	1.58 ± 0.15	5/7	1/11				
GGT1									
*AA*	61	0.43 ± 0.07	1.80 ± 0.17	14/43*	6/51**	0.032	1.000	0.011	0.009
*AG*	32	0.13 ± 0.08	1.69 ± 0.09	9/21*	4/26**	1.000	0.258		
*GG*	7	0.20 ± 0.05	2.57 ± 0.72	1/5*	0/6**	1.000	0.354		
GPRC6A									
*CC*	35	0.26 ± 0.09	1.91 ± 0.28	6/27	2/31	0.934	1.000	0.351	0.462
*CG*	54	0.38 ± 0.08	1.82 ± 0.13	16/33	7/42	0.958	1.000		
*GG*	10	0.20 ± 0.13	1.59 ± 0.23	1/9	0/10	1.000	1.000		
SPINK1									
*AA*	99	0.31 ± 0.05	1.82 ± 0.12	24/68	10/82			0.833	0.935
*AG*	1	1.00	1.30	0/1	0/1				

* *P* < 0.05 versus patients with size of cyst more than 20 mm.

** *P* < 0.05 versus patients with size of cyst more than 30 mm.

Data of EUS total score represent the sum of the score of lobularity, standing or hyperechoic foci, hyperechoic MPD and dilated side branches. Data of cyst size represent the number of patients. IPMN: intraductal papillary mucinous neoplasm, CPA1: carboxypeptidase A1, GGT1: gamma‐glutamyltransferase 1, GPRC6A: G‐protein‐coupled receptor class C Group 6 member A, SPINK1: serine protease inhibitor, Kazal type 1. *P*1 value: *P* value of EUS total score, *P*2 value: *P* value of the dilater of MPD, *P*3 value: *P* value of size of cyst more than 20 mm, *P*4 value: *P* value of size of cyst more than 30 mm. For *P*1 and *P*2 value of GGT1, *P* value of AA vs. AG is shown in the top row, AG vs. GG in the second row, GG vs. AA at the bottom. For *P*1 and *P*2 value of GPRC6A, *P* value of CC vs. CG is shown in the top row, CG vs. GG in the second row, GG vs. CC at the bottom. Statistical analysis for continuous variable of SPINK1 was not possible because the number of AG was too small. Data are represented as mean value ± SEM.

## Discussion

In this study, the major findings were 1) *CPA1* genotype was significantly associated with IPMN, 2) *GGT1* genotype was also significantly associated with EUS total score and one of worrisome features such as the size of cyst more than 20 mm and more than 30 mm.

Although the majority of IPMN will never progress toward pancreatic ductal adenocarcinoma (PDAC) and continuous surveillance is a burden that can potentially be avoided for patients and health care systems, both IPMN‐derived PDAC and concomitant PDAC have to be considered. Therefore, the identification of molecular biomarkers that can predict the risk of progression of IPMN to malignancy is critical. Taki et al. reported that KRAS and GNAS may be associated with the development of IPMN.^24^ In addition, there are not many reports of SNPs associated with the development of IPMN and cystic enlargement.^25^ In the present study, *CPA1* genotype was significantly associated with IPMN. *CPA1* is associated with a misfolding‐dependent pathway involved in the promotion of IPMN and PDAC. Additionally, we have also revealed that detection of the cleaved pattern of the C‐terminal ends of the amino acids of the apolipoprotein A2 homodimer can distinguish patients with stage I/II pancreatic cancer and associated diseases from healthy subjects.^16^, ^17^, ^18^ Similarly, *CPA1* encoding carboxypeptidase A1 in chronic pancreatitis may be associated with the risk of patients of IPMNs. In contrast, in our data, *CPA1* genotype was not linked with size of cyst in IPMN as a reprehensive worrisome feature. Gentiluomo et al. have reported that the rs1517037‐related gastrin‐releasing peptide gene and rs10094872‐related MYC proto‐oncogene were significantly associated with progression of IPMN.^26^ Further studies will be needed to compare the histological assessments of IPMN with the expression of *CPA1* genotypes to determine whether *CPA1* genotype affect with the initiation of IPMN the upregulation of the production of mucin from epithelial cells in IPMN.

Chronic pancreatitis is a known risk factor of pancreatic cancer. Gentiluomo et al. reported that the degree of chronic pancreatitis is associated with the progression of IPMN,^26^ while there are reports that pancreatic enzyme abnormalities due to microinflammation are associated with increased incidence and invasion of pancreatic cancer.^27^ In contrast, Yagi et al. have reported that pancreatic inflammation was not associated with the progression of patients with IPMN.^28^ In this study, we compared chronic pancreatitis‐related genotypes, including *GGT1*, *GPRC6A*, and *SPINK‐1*, *in* patients with IPMN. EUS score by ultrasonography, which may reflect inflammation and fibrosis of the background pancreas, showed a significant difference in EUS score in patients with IPMN for *GGT1* genotype associated with chronic pancreatitis as described in Table 7. There were some limitations for this study concerning a single institution and a single race.

In addition, the genotype of *GGT1* was significantly associated with the size of cyst more than 20 mm and 30 mm in patients with IPMN as worrisome features. *GGT1* has been reported to be involved in oxidative stress and inflammation.^29^ Smoking has been found to be involved in the malignant transformation of IPMN.^30^ In fact, Ściskalska et al. have described that smoking and *GGT1* are associated with acute pancreatitis,^31^ and Diergaarde et al. have reported an association between *GGT1* and pancreatic cancer.^14^ Then, mucin produced from IPMN was mainly MUC2 which classified as intestinal type and MUC1 was produced from malignant transformed IPMN.^32^, ^33^ This malignant transformation via sustained chronic inflammation by genotypes including *GGT1* and *SPINK‐1* may lead to MUC1 expression. Graber et al. have reported the association between inhibition of *GGT1* activity and the emergence of apoptosis.^34^ Further studies will be needed whether *GGT1* genotype may be associated with the enlargement of size of cyst through the reduction of apoptosis via KRAS activation. Considering of a relationship between cyst size increase and *GGT1* was observed, sustained inflammation and the reduction of apoptosis in IPMN may be a risk factor for the increase of cyst of IPMN. Since no SNPs have been reported for factors that increase cyst size in IPMN, further studies will be needed to clarify the precise mechanisms why *GGT1* was associated with the increase of cystic size and cystic changes in size overtime of IPMN.

Interestingly, we have previously described low levels of HDL‐Cho as a predictor of progression to early chronic pancreatitis.^35^ Although we did not find an association between *GGT1* and low HDL‐Cho in the IPMN group in our results, Jinnouchi et al. reported the involvement of HDL‐Cho and *GGT1*.^36^ However, we have previously reported that the inhibition of carboxypeptidase will lead to the reduction of apolipoprotein A2‐isoforms and HDL‐Cho levels. The low levels of apolipoprotein A2 isoforms can predict for the risk of early stage of pancreatic cancer and the disturbance of exocrine pancreatic dysfunction.^16^, ^17^, ^18^, ^20^ In contrast, in this study, *CPA1* genotype in IPMN was not significantly associated with HDL‐Cho levels. Since apolipoprotein A2 is one of the component of HDL‐Cho, the reduction of apolipoprotein A2 may be linked with the depletion of HDL‐Cho. The reduction of HDL‐Cho was reported to be associated with intrapancreatic fat deposition related to chronic pancreatitis, pancreatic dysfunction, IPMN, and pancreatic cancer.^37^, ^38^, ^39^, ^40^, ^41^ Although further studies will be needed to clarify whether *CPA1*(rs201750163) genotype was linked to the reduction of apolipoprotein A2 levels and HDL‐Cho levels, *CPA1* genotype‐related reduction of apolipoprotein A2 may be partly associated with the early stage of IPMN development. In contrast, local inflammation induced by *GGT1* genotype and adipokines derived from intrapancreatic fat deposition may be linked to the late stage of the development of IPMN.

Taken together, genotypes of *CPA1* and *GGT1* may be useful tools for the diagnosis and the predictor of worrisome features of IPMN.

## Ethics statement

The study protocol was approved by the Ethics Review Committee (M‐2023‐112) of the Nippon Medical School Hospital. We obtained the form of consent by written documents. Our study did not include minors.

## Consent to participate statement

Written informed consent was obtained from all enrolled patients.

## Data Availability

The data that support the findings of this study are available on request from the corresponding author. The data are not publicly available due to privacy reasons.

## References

[1] Ardeshna DR , Rangwani S , Cao T , Pawlik TM , Stanich PP , Krishna SG . Intraductal papillary mucinous neoplasms in hereditary cancer syndromes. Biomedicine. 2022; 10: 1–14. 10.3390/biomedicines10071475 PMC9313108 35884779

[2] Giaccherini M , Gentiluomo M , Arcidiacono PG *et al*. A polymorphic variant in telomere maintenance is associated with worrisome features and high‐risk stigmata development in IPMNs. Carcinogenesis. 2022; 43: 728–735. 35675759 10.1093/carcin/bgac051

[3] Capurso G , Crippa S , Vanella G *et al*. Factors associated with the risk of progression of low‐risk branch‐duct intraductal papillary mucinous neoplasms. JAMA Netw. Open. 2020; 3: e2022933. 33252689 10.1001/jamanetworkopen.2020.22933 PMC7705592

[4] Overbeek KA , van Leeuwen N , Tacelli M *et al*. International external validation of a stratification tool to identify branch‐duct intraductal papillary mucinous neoplasms at lowest risk of progression. United Eur. Gastroenterol J. 2022; 10: 169–178. 10.1002/ueg2.12207 PMC8911544 35199484

[5] Capurso G , Boccia S , Salvia R *et al*. Risk factors for intraductal papillary mucinous neoplasm (IPMN) of the pancreas: a multicentre case–control study. Am. J. Gastroenterol. 2013; 108: 1003–1009. 23458848 10.1038/ajg.2013.42

[6] Kuboki Y , Shimizu K , Hatori T *et al*. Molecular biomarkers for progression of intraductal papillary mucinous neoplasm of the pancreas. Pancreas. 2015; 44: 227–235. 25423558 10.1097/MPA.0000000000000253

[7] Hegyi E , Sahin‐Tóth M . Genetic risk in chronic pancreatitis: the trypsin‐dependent pathway. Dig. Dis. Sci. 2017; 62: 1692–1701. 28536777 10.1007/s10620-017-4601-3 PMC5487703

[8] Whitcomb DC , Gorry MC , Preston RA *et al*. Hereditary pancreatitis is caused by a mutation in the cationic trypsinogen gene. Nat. Genet. 1996; 14: 141–145. 8841182 10.1038/ng1096-141

[9] Witt H , Luck W , Hennies HC *et al*. Mutations in the gene encoding the serine protease inhibitor, Kazal type 1 are associated with chronic pancreatitis. Nat. Genet. 2000; 25: 213–216. 10835640 10.1038/76088

[10] Sahin‐Tóth M . Genetic risk in chronic pancreatitis: The misfolding‐dependent pathway. Curr. Opin. Gastroenterol. 2017; 33: 390–395. 28650851 10.1097/MOG.0000000000000380 PMC5549634

[11] Witt H , Beer S , Rosendahl J *et al*. Variants in CPA1 are strongly associated with early onset chronic pancreatitis. Nat. Genet. 2013; 45: 1216–1220. 23955596 10.1038/ng.2730 PMC3909499

[12] Tamura K , Yu J , Hata T *et al*. Mutations in the pancreatic secretory enzymes CPA1 and CPB1 are associated with pancreatic cancer. Proc. Natl. Acad. Sci. USA. 2018; 115: 4767–4772. 29669919 10.1073/pnas.1720588115 PMC5939087

[13] Kaune T , Ruffert C , Hesselbarth N *et al*. Analysis of GPRC6A variants in different pancreatitis etiologies. Pancreatology. 2020; 20: 1262–1267. 32859544 10.1016/j.pan.2020.08.001

[14] Diergaarde B , Brand R , Lamb J *et al*. Pooling‐based genome‐wide association study implicates gamma‐glutamyltransferase 1 (GGT1) gene in pancreatic carcinogenesis. Pancreatology. 2010; 10: 194–200. 20484958 10.1159/000236023 PMC2899150

[15] Takenaka M , Masuda A , Shiomi H *et al*. Chronic chronic pancreatitis finding by endoscopic ultrasonography in the pancreatic parenchyma of intraductal papillary mucinous neoplasms is associated with invasive intraductal papillary mucinous carcinoma. Oncology. 2017; 93: 61–68. 29258092 10.1159/000481232

[16] Honda K , Kobayashi M , Okusaka T *et al*. Plasma biomarker for detection of early stage pancreatic cancer and risk factors for pancreatic malignancy using antibodies for apolipoprotein‐AII isoforms. Sci. Rep. 2015; 5: 1–15. 10.1038/srep15921 PMC4637825 26549697

[17] Honda K , Hayashida Y , Umaki T *et al*. Possible detection of pancreatic cancer by plasma protein profiling. Cancer Res. 2005; 65: 10613–10622. 16288055 10.1158/0008-5472.CAN-05-1851

[18] Honda K , Okusaka T , Felix K *et al*. Altered plasma apolipoprotein modifications in patients with pancreatic cancer: protein characterization and multi‐institutional validation. PLoS One. 2012; 7: 1–11. 10.1371/journal.pone.0046908 PMC3466211 23056525

[19] Hayasaki A , Murata Y , Usui M *et al*. Clinical significance of plasma apolipoprotein‐AII isoforms as a marker of pancreatic exocrine disorder for patients with pancreatic adenocarcinoma undergoing chemoradiotherapy, paying attention to pancreatic morphological changes. Biomed. Res. Int. 2019; 2019: 1–12. 10.1155/2019/5738614 PMC6475573 31080824

[20] Futagami S , Agawa S , Nakamura K *et al*. Apolipoprotein A2 isoforms associated with exocrine pancreatic insufficiency in early chronic pancreatitis. J. Gastroenterol. Hepatol. 2023; 38: 1949–1957. 37501507 10.1111/jgh.16302

[21] Vendrell J , Querol E , Avileés FX . Metallocarboxypeptidases and their protein inhibitors: Structure, function and biomedical properties. Biochim. Biophys. Acta – Protein Struct. Mol. Enzymol. 2000; 1477: 284–298. 10.1016/s0167-4838(99)00280-0 10708864

[22] Ito T , Ishiguro H , Ohara H *et al*. Evidence‐based clinical practice guidelines for chronic pancreatitis 2015. J. Gastroenterol. 2016; 51: 85–92. 26725837 10.1007/s00535-015-1149-x

[23] Masao T , Carlos FC , Volkan A *et al*. International consensus guidelines 2012 for the management of IPMN and MCN of the pancreas. Pancreatology. 2012; 12: 183–197. 22687371 10.1016/j.pan.2012.04.004

[24] Taki K , Ohmuraya M , Tanji E *et al*. GNASR201H and KrasG12D cooperate to promote murine pancreatic tumorigenesis recapitulating human intraductal papillary mucinous neoplasm. Oncogene. 2016; 35: 2407–2412. 26257060 10.1038/onc.2015.294

[25] Omori Y , Ono Y , Tanino M *et al*. Pathways of progression from intraductal papillary mucinous neoplasm to pancreatic ductal adenocarcinoma based on molecular features. Gastroenterology. 2019; 156: 647–661.e2. 30342036 10.1053/j.gastro.2018.10.029

[26] Gentiluomo M , Corradi C , Arcidiacono PG *et al*. Role of pancreatic ductal adenocarcinoma risk factors in intraductal papillary mucinous neoplasm progression. Front. Oncol. 2023; 13: 1–11. 10.3389/fonc.2023.1172606 PMC10280811 37346070

[27] Roch AM , Parikh JA , Al‐Haddad MA *et al*. Abnormal serum pancreatic enzymes, but not pancreatitis, are associated with an increased risk of malignancy in patients with intraductal papillary mucinous neoplasms. Surg (United States). 2014; 156: 923–930. 10.1016/j.surg.2014.07.010 25239347

[28] Yagi Y , Masuda A , Zen Y *et al*. Pancreatic inflammation and atrophy are not associated with pancreatic cancer concomitant with intraductal papillary mucinous neoplasm. Pancreatology. 2018; 18: 54–60. 29269290 10.1016/j.pan.2017.12.007

[29] Ali SS , Oni ET , Blaha MJ *et al*. Elevated gamma‐glutamyl transferase is associated with subclinical inflammation independent of cardiometabolic risk factors in an asymptomatic population: a cross‐sectional study. Nutr. Metab. 2016; 13: 1–7. 10.1186/s12986-016-0097-7 PMC4870806 27195017

[30] Carr RA , Roch AM , Shaffer K *et al*. Smoking and IPMN malignant progression. Am. J. Surg. 2017; 213: 494–497. 28129918 10.1016/j.amjsurg.2016.10.033

[31] Ściskalska M , Ołdakowska M , Marek G , Milnerowicz H . Increased risk of acute pancreatitis occurrence in smokers with rs5751901 polymorphisms in ggt1 gene. Int. J. Med. Sci. 2020; 17: 242–254. 32038108 10.7150/ijms.38657 PMC6990886

[32] Jonckheere N , Skrypek N , Seuningen I . Mucins and pancreatic cancer. Cancers (Basel). 2010; 2: 1794–1812. 24281201 10.3390/cancers2041794 PMC3840449

[33] Castellano‐Megías VM , Ibarrola‐de Andrés C , López‐Alonso G , Colina‐Ruizdelgado F . Pathological features and diagnosis of intraductal papillary mucinous neoplasm of the pancreas. World J. Gastrointest Oncol. 2014; 15: 311–324. 10.4251/wjgo.v6.i9.311 PMC4163729 25232456

[34] Graber R , Losa GA . Apoptosis in human lymphoblastoid cells induced by acivicin, a specific gamma‐glutamyltransferase inhibitor. Int. J. Cancer. 1995; 62: 443–448. 7635570 10.1002/ijc.2910620414

[35] Agawa S , Futagami S , Watanabe Y *et al*. Combination of high‐density cholesterol level, elastic score, and severity of exocrine pancreatic dysfunction may be useful for a predictive factor for patients with early chronic pancreatitis. J. Gastroenterol. Hepatol. 2023; 38: 548–555. 36399411 10.1111/jgh.16065

[36] Jinnouchi H , Morita K , Tanaka T *et al*. Interactive effects of a common γ‐glutamyltransferase 1 variant and low high‐density lipoprotein‐cholesterol on diabetic macro‐ and micro‐angiopathy. Cardiovasc. Diabetol. 2015; 14: 1–11. 25952030 10.1186/s12933-015-0212-5 PMC4428095

[37] Skudder‐Hill L , Sequeira‐Bisson IR , Ko J , Cho J , Poppitt SD , Petrov MS . Remnant cholesterol, but not low‐density lipoprotein cholesterol, is associated with intra‐pancreatic fat deposition. Diabetes Obes. Metab. 2023; 25: 3337–3346. 37529874 10.1111/dom.15233

[38] Yamazaki H , Streicher SA , Wu L *et al*. Evidence for a causal link between intra‐pancreatic fat deposition and pancreatic cancer. A prospective cohort and Mendelian randomization study. Cell Rep. Med. 2024; 5: 101391. 38280379 10.1016/j.xcrm.2023.101391 PMC10897551

[39] Dong X , Zhu Q , Yuan C *et al*. Associations of intrapancreatic fat deposition with incident diseases of the exocrine and endocrine pancreas: a UK biobank prospective cohort study. Am. J. Gastroenterol. 2024; 119: 1158–1166. 38587286 10.14309/ajg.0000000000002792 PMC11142652

[40] Kashiwagi K , Minami K , Seino T *et al*. Pancreatic fat content may increase the risk of imaging progression in low‐risk branch duct intraductal papillary mucinous neoplasm. J. Dig. Dis. 2019; 10: 557–562. 10.1111/1751-2980.12801 31322828

[41] Sreedhar UL , DeSouza SV , Park B , Petrov MS . A systemic review of intra‐pancreatic fat deposition and pancreatic carcinogenesis. J Gastrointest Sug. 2020; 24: 2560–2569. 10.1007/s11605-019-04417-4 31749093

